# AGIA Tag System Based on a High Affinity Rabbit Monoclonal Antibody against Human Dopamine Receptor D1 for Protein Analysis

**DOI:** 10.1371/journal.pone.0156716

**Published:** 2016-06-06

**Authors:** Tomoya Yano, Hiroyuki Takeda, Atsushi Uematsu, Satoshi Yamanaka, Shunsuke Nomura, Keiichirou Nemoto, Takahiro Iwasaki, Hirotaka Takahashi, Tatsuya Sawasaki

**Affiliations:** Proteo-Science Center (PROS), Ehime University, 3 Bunkyo-cho, Matsuyama, Ehime 790–8577, Japan; Osaka University, JAPAN

## Abstract

Polypeptide tag technology is widely used for protein detection and affinity purification. It consists of two fundamental elements: a peptide sequence and a binder which specifically binds to the peptide tag. In many tag systems, antibodies have been used as binder due to their high affinity and specificity. Recently, we obtained clone Ra48, a high-affinity rabbit monoclonal antibody (mAb) against dopamine receptor D1 (DRD1). Here, we report a novel tag system composed of Ra48 antibody and its epitope sequence. Using a deletion assay, we identified EEAAGIARP in the C-terminal region of DRD1 as the minimal epitope of Ra48 mAb, and we named this sequence the “AGIA” tag, based on its central sequence. The tag sequence does not include the four amino acids, Ser, Thr, Tyr, or Lys, which are susceptible to post-translational modification. We demonstrated performance of this new tag system in biochemical and cell biology applications. SPR analysis demonstrated that the affinity of the Ra48 mAb to the AGIA tag was 4.90 × 10^−9^ M. AGIA tag showed remarkably high sensitivity and specificity in immunoblotting. A number of AGIA-fused proteins overexpressed in animal and plant cells were detected by anti-AGIA antibody in immunoblotting and immunostaining with low background, and were immunoprecipitated efficiently. Furthermore, a single amino acid substitution of the second Glu to Asp (AGIA/E2D) enabled competitive dissociation of AGIA/E2D-tagged protein by adding wild-type AGIA peptide. It enabled one-step purification of AGIA/E2D-tagged recombinant proteins by peptide competition under physiological conditions. The sensitivity and specificity of the AGIA system makes it suitable for use in multiple methods for protein analysis.

## Introduction

Polypeptide tag technology, based on the interaction between a monoclonal antibody (mAb) and its epitope peptide, is an essential tool for protein analysis [1–4]. Commercially available peptide tag systems such as the FLAG [5,6], HA [7], MYC [8], and V5 [9] tags are widely used in cell biology and biochemical analysis of proteins. Although these tags are useful in current biological studies, several aspects of these tags require further improvement. First, some tag antibodies cross-react with other proteins resulting in increased background noise in immunoblotting, immunoprecipitation, and immunostaining. The level of background noise depends on the specificity and affinity of the antibody for the tag. Second, post-translational modification (PTM) of tag sequences can occur. Recent proteomics approaches have reported that amino acids such as Ser, Thr, and Tyr, or Lys are phosphorylated or ubiquitinated in eukaryotic cells, respectively [10–13]. In addition, Tyr residue can be sulfated in the trans-Golgi network [14]. If a tag sequence contains these residues, it is possible that they will be modified by cellular enzymes. To our surprise, all commercially available tag sequences include at least one of the four commonly modified amino acids: FLAG (DYKDDDDK), HA (YPYDVPDYA), MYC (EQKLISEEDL), and V5 (GKPIPNPLLGLDST), where underlines represent the amino acids in question. These residues may contribute to improve hydrophilicity or antigenicity, as Hopp et al. intentionally inserted Tyr and Lys in FLAG tag sequence [5]. However, it is also possible that PTMs occur on these residues. Although there are only few examples published, for example, Schmidt et al. reported that when FLAG tag is fused to secreted protein and expressed in insect cell system, Tyr residue of FLAG tag is highly sulfated, and reactivity of anti-FLAG antibody toward sulfated FLAG tag decreases drastically [14]. The possibility cannot be ruled out that other PTM also compromised the tag system or affect the fate of the tagged protein in the cell. Not only tag performance, PTM may change the behavior, localization, and stability of tag-fusion recombinant proteins, or may affect the results of cell biology and biochemical analysis. Therefore, development of a tag system that excludes these four amino acids is desirable.

Recently, rabbit antibodies have attracted much attention because of their extremely high specificity and affinity [15–17]. However, mAb isolation from rabbit is very difficult because usual hybridoma techniques cannot be used on rabbit leukocytes. Therefore, peptide tag technology based on a rabbit mAb has not been reported to date. However, recent innovations in antibody technology, such as the development of improved fusion partners or technologies for cloning immunoglobulin cDNA, have allowed more efficient production of rabbit mAbs [18,19]. We isolated six high affinity rabbit mAb clones against the human dopamine receptor D1 (DRD1) using immunospot array assay on a chip (ISAAC) technology [17]. We focused on mAb clone Ra48, which had the highest affinity (K_d_ = 0.86 × 10^−10^ M) of the six rabbit anti-DRD1 antibodies, and which was suitable for immunoblotting, immunoprecipitation, and immunostaining. We propose that the Ra48 antibody and its epitope sequence are suitable for developing a high performance affinity tag system with the first rabbit mAb. Here, we report the development of the “AGIA tag” system, and demonstrate its performance in cell biology and biochemical analysis.

## Results

### Expression of DRD1 Is Limited in Cell Lines and Several Tissues

**Fig 1:**
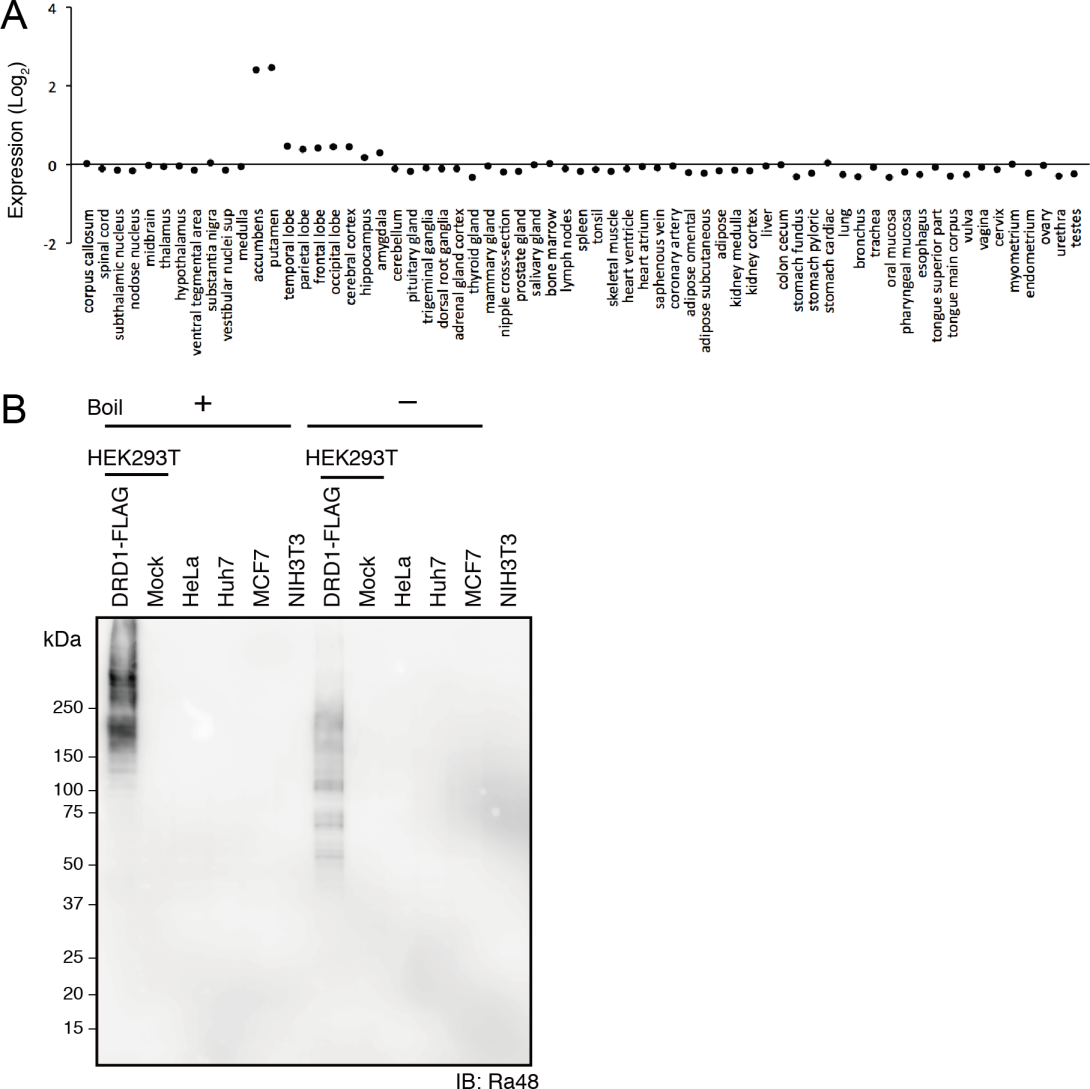
Limited expression of DRD1 in human tissues and cell lines. (A) Expression of human DRD1 mRNA in human tissues. Expression data was obtained from the COXPRESSdb (http://coxpresdb.jp/) [48]. The probe set was 214652_at. (B) Cross-reactivity of anti-DRD1 Ra48 antibody against endogenous proteins in mammalian cultured cells. Cell extracts were collected from DRD1-FLAG overexpressed HEK293T cells and a number of human and mouse cultured cell-lines. 0.4 μg/mL of anti-DRD1 Ra48 antibody was used as primary antibody, and anti-rabbit IgG-HRP was used as secondary antibody.

DRD1 is a receptor of the neurotransmitter dopamine, which is involved in signal transduction at synapses in the brain [20,21]. We predicted that the expression of DRD1 is limited to the central nervous system and is suppressed in other cells and tissues. Indeed, microarray data showed that expression of DRD1 mRNA was low in almost all tissues and organs except for accumbens and putamen as limited regions of the brain (Fig 1A). Next, we used immunoblotting to compare the reactivity of Ra48 mAb against five popular mammalian cell lines. Ra48 mAb clearly visualized DRD1-FLAG protein overexpressed in HEK293T cells around 50–250 kDa (Fig 1B, without heat denature). The estimated size of DRD1-FLAG was around 50 kDa, thus we estimated that the high molecular weight-multiple bands were derived from multimerization or post-translational modification of DRD1. In the left half of the panel, where heat-denatured samples were applied, overexpressed DRD1 was seen as smear band due to aggregation. Ra48 mAb did not detect any clear extra bands in lysates from HEK293T, HeLa, Huh7, MCF7, and NIH3T3 cells. These results indicate that Ra48 mAb specifically recognized the DRD1 epitope with excellent signal-noise level. This specificity is advantageous in cell biology.

### Determination of the Minimal Amino Acid Sequence Recognized by Ra48 mAb

**Fig 2:**
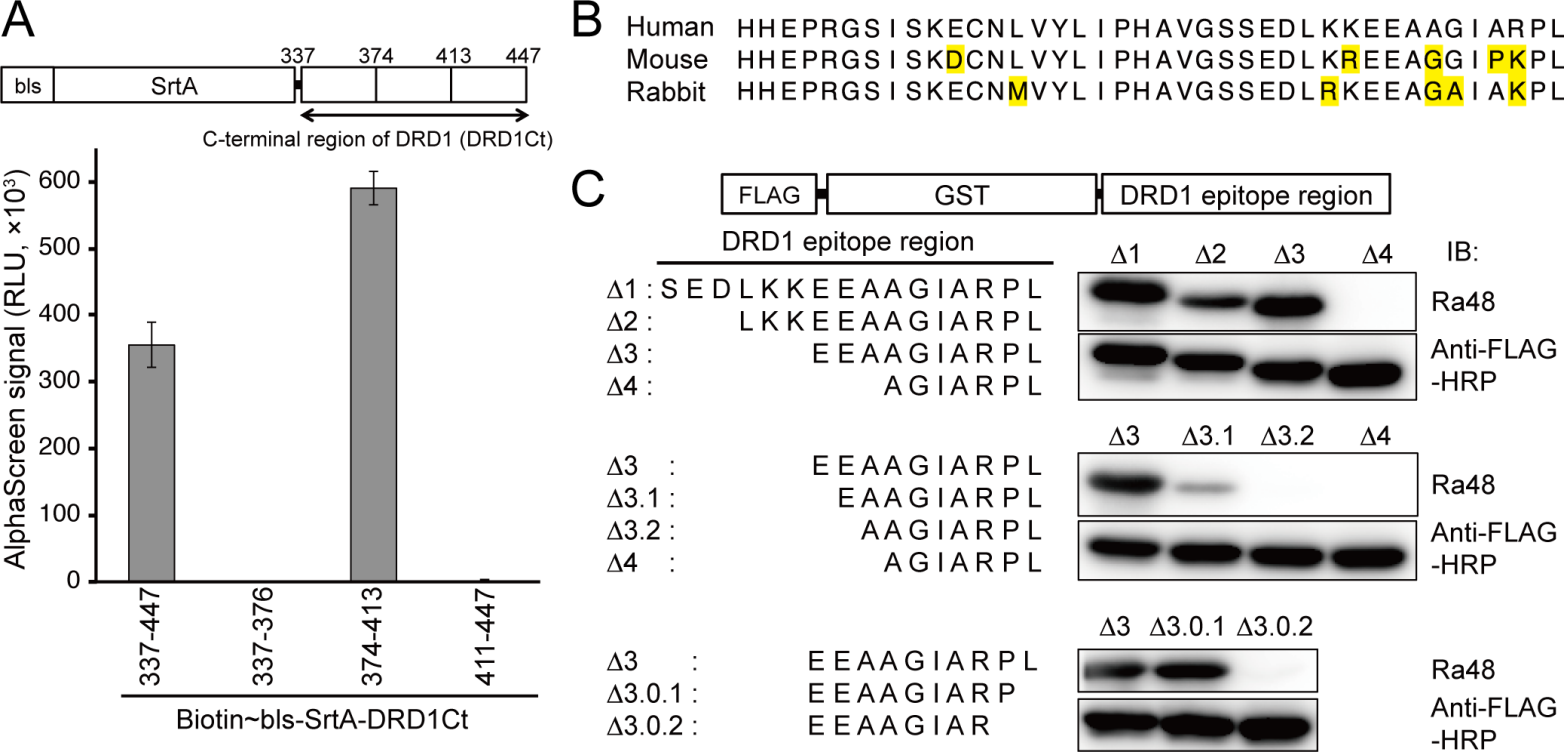
Epitope mapping of Ra48 mAb. (A) Identification of epitope region using DRD1 C-terminal fragments. The full (337–447) or partial fragments (337–376, 374–413, and 411–447) of the C-terminal cytoplasmic region of human DRD1 were fused to the C-terminus of bls-SrtA protein and synthesized using wheat germ cell-free system. Binding between the fusion protein and Ra48 antibody was detected by AlphaScreen. (B) Comparison of amino acid sequences between human DRD1 (374–413), mouse Drd1 (374–413), and rabbit DRD1 (376–415). Yellow denote the amino acids different from ones of human DRD1. (C) Immunoblot analysis of deletion mutants to identify the minimum Ra48 epitope sequence.

Our previous study showed that Ra48 mAb does not react with a swapped DRD1 mutant, whose C-terminal intracellular region (337–447) is swapped with the region of another class A GPCR; in addition, Ra48 mAb binds to a recombinant protein fragment, biotin-sortaseA-DRD1 (337–447) [17]. These data suggest that the Ra48 epitope is in this region. To identify the epitope, the C-terminus was divided into three segments, and binding between biotin-sortaseA-DRD1 fragment and Ra48 mAb was detected using AlphaScreen [22]. Ra48 mAb recognized the central region (374–413) (Fig 2A). In addition, Ra48 mAb did not react to mouse DRD1 [17]. Comparing human, mouse and rabbit DRD1 sequences in this region, we found that SEDLKKEEAAGIARPL (398–413) contains several unconserved residues (Fig 2B). In fact, Ra48 mAb recognized a fragment spanning amino acids 398–413, fused to the C-terminal of FLAG-glutathione-*S*-transferase (GST) (upper panel in Fig 2C). We deleted every three amino acids from the N terminal of the fragment, and examined Ra48 reactivity. Ra48 recognized EEAAGIARPL (Δ3) but did not bind to AGIARPL (Δ4). Next, a single deletion from the N-terminal of Δ3 fragment was generated, which confirmed that the N terminal Glu-Glu was necessary for antibody binding (middle panel). Deletions in the C-terminal end of the Δ3 fragment were generated in the same way (lower panel). Finally we identified the minimum epitope sequence of Ra48 mAb as nine amino acids (EEAAGIARP) corresponding to human DRD1 404–412. We named the EEAAGIARP sequence “AGIA tag,” which came from the four amino acids in the center of the epitope sequence. Hereafter, we refer to the Ra48 mAb as anti-AGIA antibody. The AGIA tag sequence did not contain any of the most common eukaryotic post-translationally modified amino acid residues (Ser, Thr, Tyr, or Lys). Except for His tag, AGIA tag is the first short peptide tag, which does not contain these four residues to the best of our knowledge.

### Biochemical Characterization of AGIA Tag

**Fig 3:**
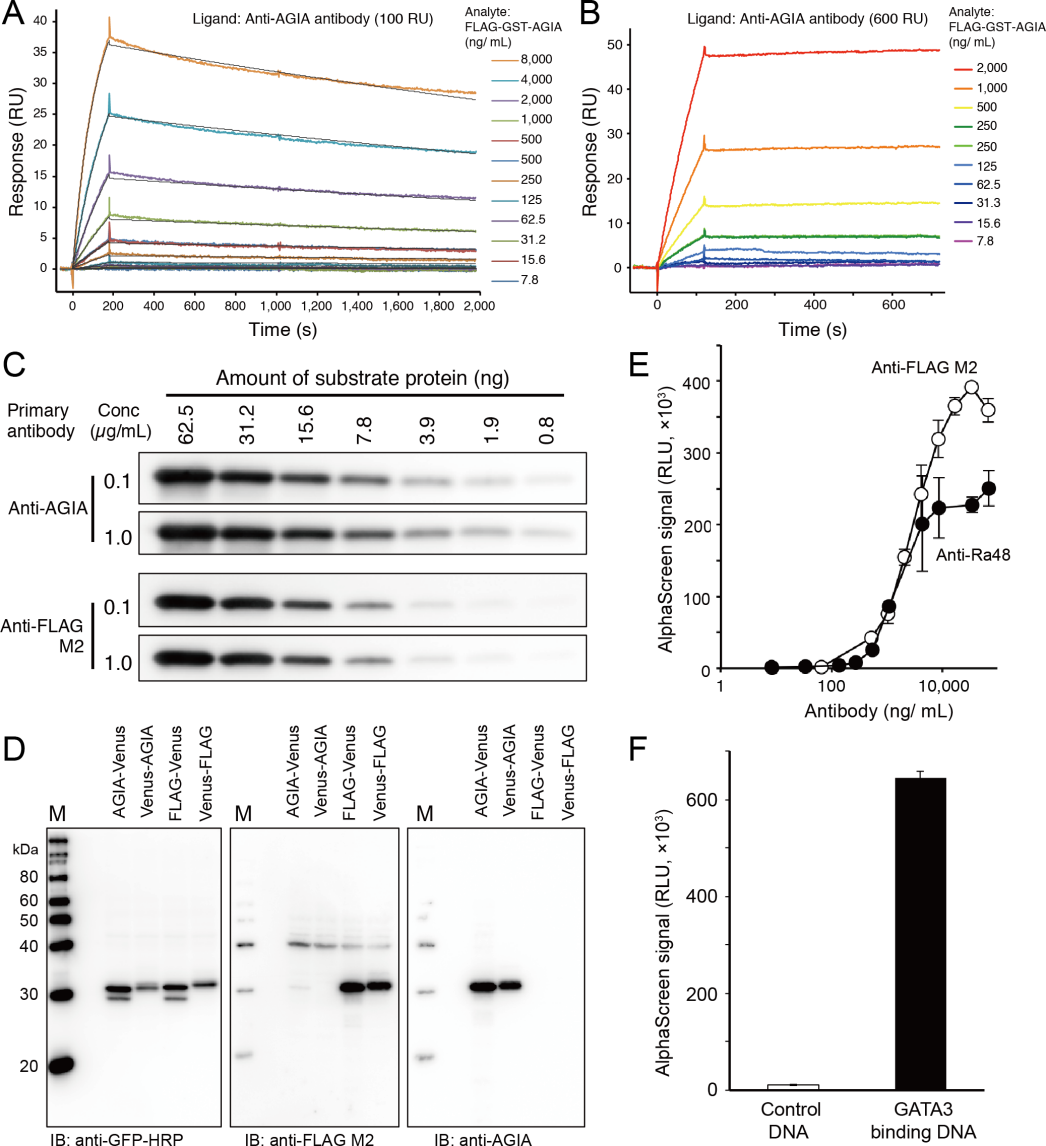
Biochemical characterization of AGIA tag. (A) Kinetics assay of AGIA tag and anti-AGIA antibody. Anti-AGIA antibody was captured on a protein G-immobilized Biacore sensorchip at 100 RU. Purified FLAG-GST-AGIA protein was then injected for 180 sec as analyte. Black lines represent a global fit of a 1:1 interaction model to each kinetic data set. (B) Stable capturing of AGIA-tagged protein by anti-AGIA antibody. Anti-AGIA antibody was captured on a protein G-immobilized Biacore sensorchip at 600 RU. Purified FLAG-GST-AGIA protein was then injected as analyte for 180 sec at 30 μL/min. (C) High sensitivity of AGIA tag in immunoblotting. FLAG-GST-AGIA protein was synthesized by cell-free system and purified using glutathione sepharose. Concentration of the purified protein was determined by extinction coefficient method [46]. Decreasing amounts of purified FLAG-GST-AGIA were applied to the immunoblot and detected by the antibodies at the concentrations shown. (D) Specific detection of recombinant AGIA-tagged protein by anti-AGIA antibody. AGIA (AGIA-Venus or Venus-AGIA) or FLAG tag (FLAG-Venus or Venus-FLAG) fused Venus proteins were synthesized by cell-free system and applied to immunoblotting. (E) Performance of AGIA tag in protein-protein interaction assay. FLAG- or AGIA-tagged p53 was mixed with biotin-Mdm2, and interaction between the proteins was detected with AlphaScreen using various concentrations of anti-FLAG M2 or anti-AGIA antibodies. (F) Performance of AGIA tag in protein-DNA interaction assay. AGIA-tagged GATA3 protein was mixed with biotinylated oligonucleotides containing GATA3 binding sequence, and the binding was detected with AlphaScreen.

To investigate the affinity of the AGIA tag system, which consisted of the AGIA tag and the anti-AGIA antibody, FLAG-GST-AGIA recombinant protein was synthesized and purified using glutathione sepharose. We attempted to determine affinity between purified FLAG-GST-AGIA protein and anti-AGIA antibody by using surface plasmon resonance (SPR). When 100 RU of anti-AGIA antibody was captured on a sensorchip, Kinetic analysis from100 RU yielded a K_D_ value = 4.90 × 10^−9^ M (K_a_ = 3.18 × 10^4^ 1/Ms, K_d_ = 1.56 × 10^−4^ 1/s) (Fig 3A). It should be note that when slightly higher amount of anti-AGIA antibody (600 RU) was used, dissociation of AGIA-tagged protein was hardly observed due to the re-binding of tagged protein (Fig 3B). Rebinding as shown in Fig 3B indicates the mAb would work well for column affinity chromatography. Applications such as western blotting, immunoprecipitation, and ELISA also can benefit from the avidity. Indeed, Fig 3C showed remarkable detection sensitivity of the AGIA tag. Anti-AGIA antibody clearly detected as low as around 1.9 to 0.8 ng of the target protein, which is difficult to be detected by anti-FLAG antibody.

Next, we tested whether the position of the AGIA tag in recombinant proteins influences the detection. The AGIA tag was fused to the N-terminus or the C-terminus of Venus fluorescent protein [23]. FLAG-Venus and Venus-FLAG proteins were also constructed and used as controls. These proteins were synthesized in a wheat germ cell-free system, and tagged protein in a crude translation reaction was detected by immunoblotting. Both N-terminal and C-terminal AGIA-tagged Venus proteins were clearly detected by anti-AGIA antibody (Fig 3D), indicating that the position of the AGIA tag does not affect interactions between the tag and the detection antibody. In addition, the background signal of anti-AGIA antibody detection in wheat germ cell-free extract was quite low level, similar to the signal in lysates of mammalian cell-lines (Fig 1B).

We and other groups have conducted AlphaScreen protein-protein interaction assays using the FLAG tag system [24–27], in which FLAG-tagged bait protein was captured by protein-A conjugated acceptor bead via anti-FLAG antibody. Performance of the AGIA tag system in AlphaScreen assays was evaluated using the interaction between Mdm2 and p53 as a model [28]. Similar to previous results with the FLAG tag system, interaction between AGIA-p53 and biotin-Mdm2 was successfully detected using anti-AGIA antibody (Fig 3E). The luminescent signal increased according to the anti-AGIA antibody concentration; however, it was saturated at 8,000 ng/mL. When FLAG tag and anti-FLAG M2 antibodies were used, the luminescence signal increased continuously until the antibody concentration reached 30,000 ng/mL. Next, interaction between DNA and GATA3 protein was also detected using the AGIA tag system. GATA3 is known as a transcription factor that binds to DNA [29]. N-terminal AGIA-tagged GATA3 protein was synthesized using a wheat cell-free system and mixed with a biotin-labeled, GATA3 binding oligonucleotide or with negative control DNA. A specific, luminescent signal was detected when GATA3 protein was reacted with GATA3 binding DNA, but not when reacted with negative control DNA (Fig 3F), indicating that the AGIA tag can be used to detect DNA-protein interaction by AlphaScreen technology.

### Performance of AGIA Tag System in Cell Biology

**Fig 4:**
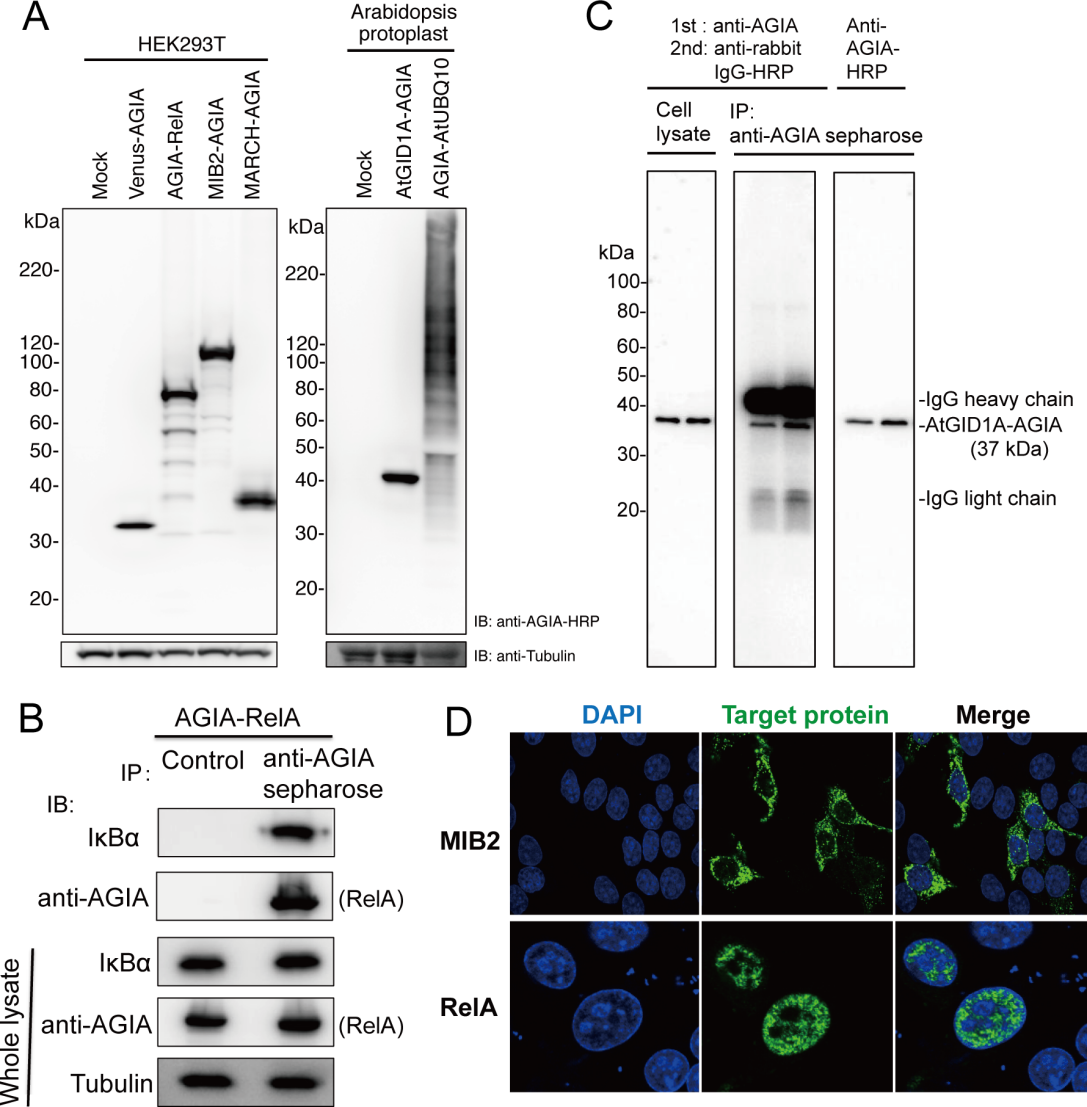
Performance of AGIA tag in Cell biological analysis. (A) Detection of tagged proteins overexpressed in animal (HEK293T, left panel) or plant cells (Arabidopsis protoplast, right). (B) Co-immunoprecipitation. AGIA-RelA was overexpressed in HEK293T cells (Whole lysate). Immunoprecipitation was performed using anti-AGIA antibody conjugated sepharose (anti-AGIA sepharose). Control experiment was performed using protein G sepharose and normal rabbit IgG (Control). Immunoprecipitated proteins were detected with IκBα specific antibody and anti-AGIA antibody. (C) Performance of anti-AGIA-HRP in immunoprecipitation. AtGID1A-AGIA protein was expressed in Arabidopsis protoplast (left panel). Lysate from protoplast was immunoprecipitated using anti-AGIA antibody sepharose (anti-AGIA sepharose). Subsequently, immunoblotting was conducted using a combination of anti-AGIA and anti-rabbit IgG-HRP antibodies (center) or anti-AGIA-HRP antibody (right), with duplicate sample loading. (D) Immunostaining. MIB2-AGIA and AGIA-RelA were expressed in HeLa cells. Tagged proteins were visualized using anti-AGIA primary antibody with anti-rabbit IgG-Alexa488 secondary antibody (green). Nucleus was stained by DAPI (blue).

To evaluate the AGIA tag system using cell extract analysis, a series of genes, including Venus, RelA, MIB2, MARCH8, and the Arabidopsis genes AtGID1A and AtUBQ10, were expressed in mammalian or Arabidopsis cells with a AGIA tag fused to their N- or C-terminus. All AGIA-tagged proteins were detected by immunoblotting (Fig 4A), indicating that AGIA tag system is effective in both mammalian and plant cells. Because AtUBQ10 is an ubiquitin protein, the smear bands in the lane (AGIA-AtUBQ10) suggest that many endogenous proteins were ubiquitinated with AGIA-tagged ubiquitin. In addition, the background signal was minimal when using lysates from either Arabidopsis cell lysates or mammalian cells, suggesting that the AGIA tag system is suitable for protein analysis of plant lysates.

Next, we tested the performance of AGIA tag system in immunoprecipitation assays. Unlike immunoblot analysis, immunoprecipitation requires that an anti-tag antibody recognizes the epitope sequence on a target protein that has retained its native tertiary structure. We used RelA and IκBα, a well-known interaction pair [30], to validate the AGIA tag system in immunoprecipitation assays. Anti-AGIA antibody was immobilized on sepharose beads using amine-coupling chemistry (anti-AGIA antibody sepharose). We confirmed capture ability of anti-AGIA antibody sepharose by using GST purified and quantified FLAG-GST-AGIA protein. A portion of anti-AGIA antibody sepharose was mixed with excess amount of FLAG-GST-AGIA protein, and incubated for 1h at 4°C. Then the sepharose was separated from the solution by column filtration, and remaining protein concentration in the solution was assayed. Considering the difference between negative control, in which buffer was added instead of the sepharose slurry, we determined the amount of the protein captured by the sepharose, and the capacity of anti-AGIA sepharose was estimated as 0.67 ± 0.11 mg protein/mL sepharose (n = 3). We immobilized 2 mg of anti-AGIA antibody to 1 mL sepharose. Considering the difference of molecular mass between antibody and tagged GST and bivalent binding of antibody, theoretical maximum capture amount of FLAG-GST-AGIA toward the sepharose is 0.8 mg/mL. Therefore we thought that the capacity estimated as above was reasonable. AGIA-tagged RelA was expressed in HEK293T cells, and the cell lysate was subsequently subjected to immunoprecipitation with anti-AGIA antibody sepharose. Subsequent immunoblot analysis demonstrated that the endogenous IκBα was successfully co-immunoprecipitated with AGIA-RelA (Fig 4B). In addition, we introduced a method for antibody detection during immunoprecipitation by using horseradish peroxidase (HRP) conjugated anti-AGIA antibody (anti-AGIA-HRP antibody). In immunoprecipitation, the immobilized heavy and light chains of the capture antibody are often cross-reacted with secondary antibodies, which may interfere with the detection of target proteins with similar molecular weight. Indeed, when immunoprecipitated sample was subjected to immunoblotting and detected by combination of anti-AGIA antibody and anti-rabbit IgG-HRP secondary antibody, undesired bands derived from immunoglobulin were observed (Fig 4C, middle panel). Especially band of the heavy chain was obvious. This problem was solved by using an HRP conjugated primary antibody. By using anti-AGIA-HRP antibody, the AtGID1A-AGIA protein (40 kDa) was successfully immunoprecipitated from Arabidopsis cell lysate, and cross-reacting bands corresponding to the capture antibody disappeared (Fig 4C, right panel). These results suggested that the AGIA tag system is a suitable tool for immunoprecipitation and co-immunoprecipitation analyses.

Determining the cellular localization of a target protein, using a method such as immunostaining, is essential for understanding of biological function of the protein, and a tag system with high sensitivity and low background is crucial. To test whether the AGIA tag system is suitable for immunostaining analysis, MIB2 and RelA were expressed in HeLa cells as AGIA-tagged fusion proteins. After fixation and permeabilization, AGIA-tagged proteins were visualized with anti-AGIA antibody and a fluorescently labeled secondary antibody. Localization of MIB2-AGIA and RelA-AGIA were identical to the previously reported localization of MIB2 and RelA (Fig 4D) [31,32], with minimal background staining. These data imply that the AGIA tag system is suitable for immunostaining.

### Single Amino Acid Substitution Enabled Competitive Dissociation of AGIA Tag

**Fig 5:**
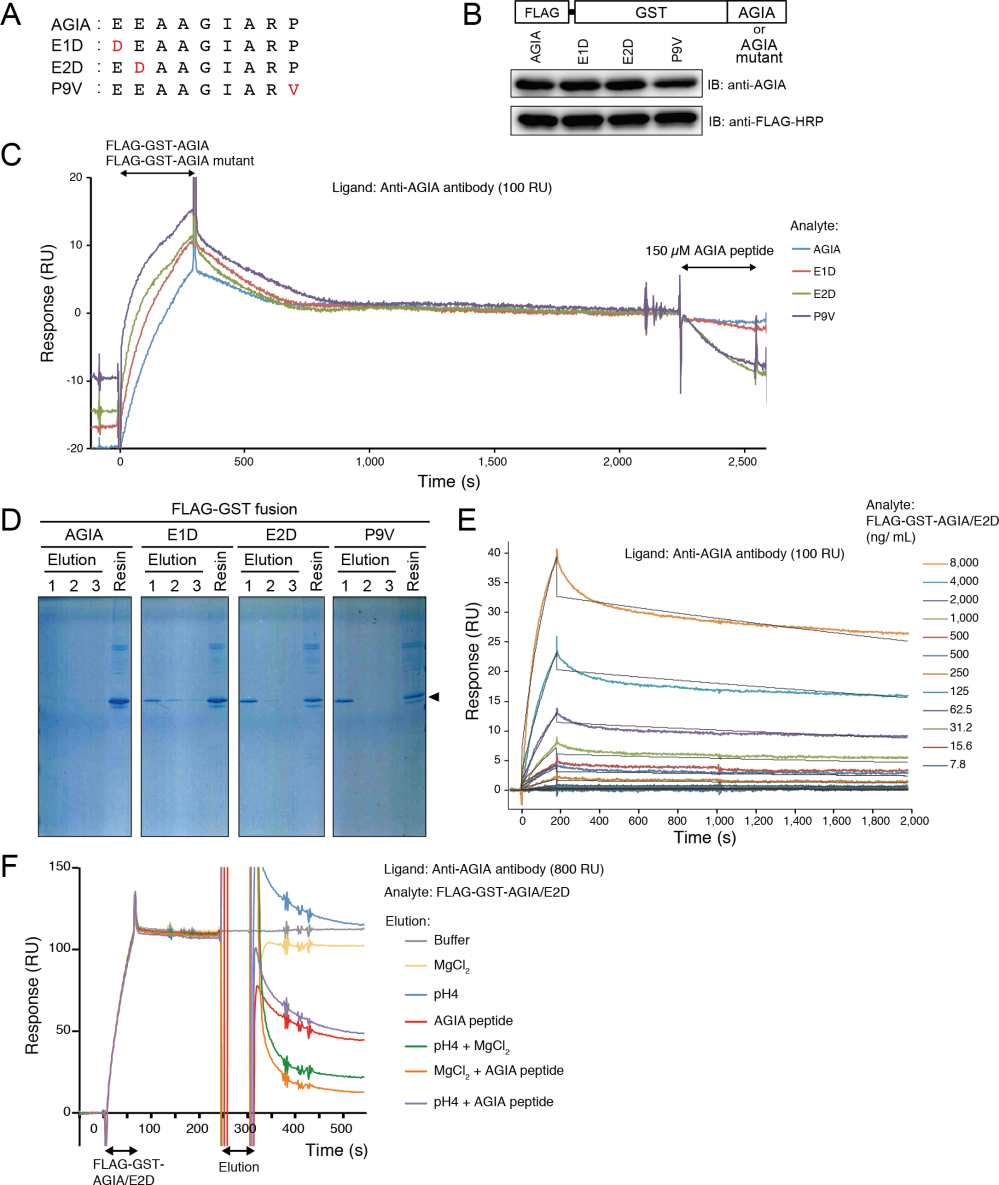
Development of AGIA/E2D tag. (A) Amino acid substitution of AGIA tag sequence. Red characters indicate substituted residues. (B) Immunoblot of proteins fused with AGIA mutants. FLAG-GST-AGIA / AGIA mutants were subjected to western blotting. (C) Amino acid substitution in the AGIA tag enabled competitive dissociation. Anti-AGIA antibody was captured on a protein G-immobilized Biacore sensorchip at 100 RU. FLAG-GST-AGIA/AGIA mutant was injected for 300 sec, and dissociation was observed for 1,800 sec. Then, 150 μM AGIA peptide was injected for 300 sec. Blue, wild-type AGIA: red, AGIA/E1D; green, AGIA/E2D; purple, AGIA/P9V. (D) Competitive elution of AGIA mutants by AGIA peptide. Six hundred μL of cell-free synthesized FLAG-GST-AGIA/mutant was mixed with 20 μL anti-AGIA sepharose. After washing with 200 μL HBS three times, proteins were eluted 3 times by 200 μL of 150 μM AGIA peptide (Elution). After elution, the resin was boiled in SDS-PAGE sample buffer for 10 min (Resin). Fractions were applied to SDS-PAGE and CBB staining. (E) Kinetics assay of AGIA/E2D tag. Anti-AGIA antibody was captured on a protein G-immobilized Biacore sensorchip at 100 RU. Purified FLAG-GST-AGIA/E2D protein was injected for 180 sec as analyte. Black lines represent a global fit of a 1:1 interaction model to each kinetic data set. (F) Elution condition of AGIA/E2D tag. Anti-AGIA antibody was captured on a protein G immobilized Biacore sensorchip at 800 RU, and FLAG-GST-AGIA/E2D was injected for 60 sec. The sensorchip was then treated by following elution solutions; gray, HBS-EP+ buffer; yellow, 2M MgCl_2_; blue, 100 mM sodium acetate buffer pH4.0, red, 150 μM AGIA peptide; green, 100 mM sodium acetate buffer pH4.0 and 2M MgCl_2_; orange, 2M MgCl_2_ and 150 μM AGIA peptide; purple, 100 mM sodium acetate buffer pH4.0 and 150 μM AGIA peptide.

The biggest advantage of AGIA tag system is its high affinity and stable binding, however, it may narrow the range of its application. For example, purification system is difficult to be developed by using high affinity tag. Indeed, once an AGIA-tagged protein bound to anti-AGIA antibody, it was difficult to cancel the binding by adding competitive AGIA peptide (Fig 5C, indicated by blue line). In order to use the AGIA tag for protein purification, it was necessary to increase dissociation constant. We hypothesized that the two glutamic acids (E) at the N terminus and the proline (P) at the C terminus of AGIA tag sequence (Fig 5A) are important for binding, because deletion of these three residues drastically decreased the binding between the sequence and anti-AGIA antibody (Fig 2C). We substituted Glu with Asp (E1D and E2D) and Pro with Val (P9V) (Fig 5A). These substitute mutations were introduced into the FLAG-GST-AGIA protein. The amino acid substitutions did not have apparent effect on the immunoreactivity with anti-AGIA tag antibody (Fig 5B, upper panel), nor did it affect the production of the tagged protein (Fig 5B, lower panel). We examined binding and competitive elution of these mutant tags by SPR (Fig 5C). Competitive elution was conducted using 150 μM of wild-type AGIA peptide, which consisted of AGIA tag sequence and additional residues at the N-terminus and C-terminus (EKKEEAAGIARPLEK, underline is minimum AGIA tag sequence). The additional residues contributed to improve solubility of the resultant peptide. These amino acid substitutions did not show considerable difference in association constant with wild-type AGIA tag. Once tagged proteins were captured by antibody, they were hardly released at least for 30 min, and dissociation constant of each mutant tags was not much different from wild-type AGIA tag. However, when wild-type AGIA peptide was injected, E2D- or P9V-tagged protein was efficiently dissociated, respectively. On the other hand, wild type and E1D were still stably captured by anti-AGIA antibody. We examined the small-scale batch purification of these mutants to confirm the SPR result. Cell-free synthesized FLAG-GST-AGIA proteins with wild type and mutant AGIA sequences were captured by anti-AGIA antibody sepharose. After washing, the resin was treated with 150 μM AGIA peptides, and eluted protein and non-eluted protein were visualized by SDS-PAGE and Coomassie brilliant blue (CBB) staining (Fig 5D). Wild-type AGIA tagged protein was not eluted by AGIA peptide and remained on the sepharose. In contrast, mutant AGIA-tagged proteins were eluted by AGIA peptide. E2D and P9V were eluted more efficiently compared with E1D. The results well agreed with SPR results. Considering the efficacy of the competitive elution in SPR, we selected the E2D mutant (AGIA/E2D) for further analysis.

Kinetics assay determined that K_D_ value between AGIA/E2D tagged protein and anti-AGIA antibody was 7.03 × 10^−9^ M (K_a_ = 2.08 × 10^4^ 1/Ms, K_d_ = 1.46 × 10^−4^ 1/s) (Fig 5E). Without competitive peptides, affinity of AGIA/E2D was not much decreased compared with one of wild-type AGIA tag (Fig 3A). Next we examined elution of AGIA/E2D in various conditions (Fig 5F). Elution efficacy of 150 μM AGIA peptide was superior to ones of weak acid (pH 4.0) and high concentration of magnesium ion (2M MgCl_2_). Among the conditions examined, the combination of AGIA peptide and MgCl_2_ was the most efficient eluate (Fig 5F, orange line). Optimization of elution condition revealed that a simple elution by AGIA peptide is useful for sample preparation for biochemical assay, in which mild condition is desirable to keep the activity of target protein.

### Protein Purification by Using AGIA Tag

Protein purification is critical step in the biochemical analysis of protein. Using an affinity tag system, highly amounts of purified protein can be obtained with fewer purification steps. For example, the FLAG tag system is widely used for protein purification, in which purified protein is eluted competitively by excess amount of FLAG tag peptide [6,33]. We attempted to develop a protein purification system based on the AGIA and AGIA/E2D tags.

**Fig 6:**
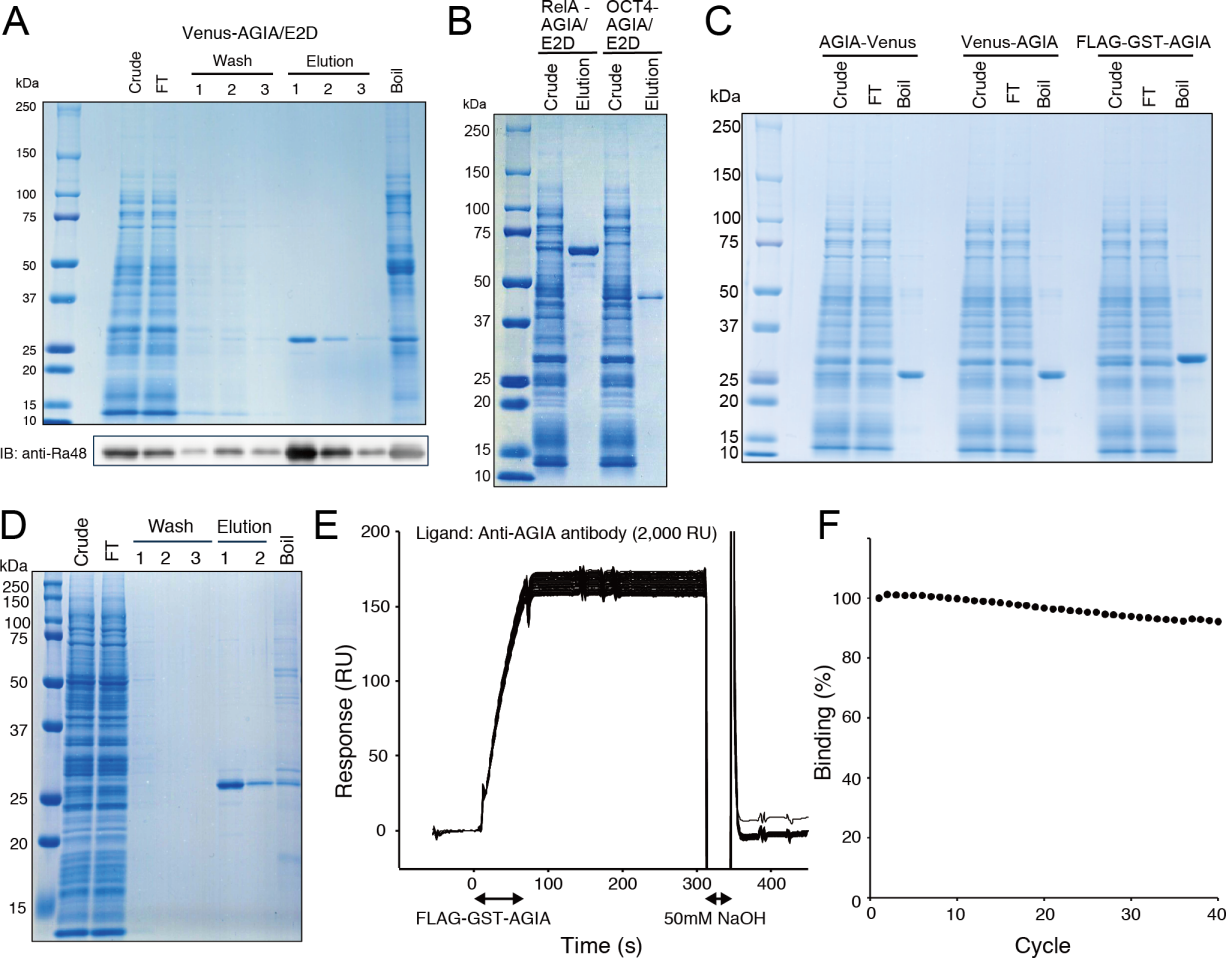
Protein purification using AGIA and AGIA/E2D tag. (A) Protein purification by using AGIA/E2D tag and peptide elution. Six mL of cell-free synthesized Venus-AGIA/E2D was mixed with 200 μL anti-AGIA sepharose. After washing, elution was performed by using 150 μM AGIA peptide. CBB staining image and immunoblotting result were shown in upper and lower panel, respectively. (B) OCT4-AGIA/E2D and RelA-AGIA/E2D were purified by anti-AGIA sepharose and AGIA peptide elution. (C) Capturing AGIA tagged protein without linker sequence. AGIA tag was directly fused to target proteins, and. cell-free synthesized. AGIA-tagged proteins were mixed with anti-AGIA sepharose, and captured proteins were eluted by boiling in SDS-PAGE sample buffer. (D) Protein purification by using AGIA tag and TEV cleavage. Venus-TEV-AGIA protein was cell-free synthesized and mixed with anti-AGIA sepharose. After washing, the resin was mixed with TEV solution and incubated for 3 h at 16°C. (E, F) Regeneration of anti-AGIA antibody. Anti-AGIA antibody was immobilized on a sensorchip CM5 by amine coupling. After 60 sec injection of FLAG-GST-AGIA protein, the chip surface was regenerated by 50 mM NaOH. Panel E showed a sensorgram of 40-cycle repeat of binding and regeneration. Binding level of FLAG-GST-AGIA in each cycle was plotted in panel F.

As described above, AGIA/E2D can be dissociated from anti-AGIA antibody by using AGIA peptide. We demonstrated the performance of protein purification by competitive elution of AGIA peptide. Venus-AGIA/E2D protein was purified successfully to a clear, single band with high purity (Fig 6A, upper panel). Immunoblotting showed that around one third of the tagged protein still bound to the sepharose after peptide elution (Fig 6A, lower panel). The elution efficacy would be improved by adding MgCl_2_ to elution solution (Fig 5F). We also confirmed that OCT4-AGIA/E2D and RelA-AGIA/E2D were also successfully purified as a single band (Fig 6B). These data demonstrated the high performance of the single step purification using AGIA/E2D tag.

We also examined whether AGIA tag directly fused to the target proteins is recognized anti-AGIA antibody. In this study ORFs of the target proteins were inserted into expression vectors containing AGIA tag sequence using Gateway system (see method and S1 Table). Thus, 8 or 9 amino acid sequence, which derived from *att*B1 or *att*B2 sequence of Gateway system, was inserted between AGIA tag and target protein. We removed Gateway *att* sequences from expression plasmids of AGIA-tagged proteins by inverse PCR, and prepared Venus and GST directly fused with AGIA tag at N or C terminus. As the result, these proteins were successfully captured by anti-AGIA antibody sepharose (Fig 6C), indicating that linker sequence is not required for AGIA tag purification. It is advantageous when shorter tag is desirable for structural or functional analysis of target protein.

In addition to the competitive elution, we also attempted on-bead TEV cleavage purification using wild-type AGIA tag. TEV cleavage site was inserted between Venus and AGIA tag, and cell-free synthesized Venus-TEV-AGIA protein was captured by anti-AGIA antibody sepharose. After washing, the sepharose was treated by TEV protease at 16°C for 3h. As the result, Venus was successfully cleaved and recovered (Fig 6D). Anti-AGIA antibody can tightly capture an AGIA tagged protein (Fig 3B), and the captured protein can be washed efficiently by various solutions such as high salt or detergents without risk of dissociation. It contributes to improve the purity of cleaved target proteins.

Finally, we report an efficient regeneration condition of anti-AGIA antibody. We conducted regeneration test of anti-AGIA antibody by SPR. Anti-AGIA antibody was immobilized on a sensorchip by amine coupling. FLAG-GST-AGIA protein was injected, and the chip surface was treated by various solutions. We found that 50 mM NaOH efficiently removed captured AGIA tagged protein (Fig 6E). After 40-cycle repeat of binding and regeneration, the loss of capture level was only 8% (Fig 6F). This data implies the possibility that anti-AGIA antibody conjugated sepharose can be regenerated by 50 mM NaOH and use repeatedly.

## Discussion

Short peptide tags are a basic tool for detection, purification and immobilization of proteins of interest [1–4], and the applications of these tags are widespread in cell biology and biochemical assays, including immunoblotting, immunoprecipitation, immunostaining, ELISA, and AlphaScreen. Sequence length of conventional well-used short tags, such as FLAG, MYC, HA and V5 tags, range from eight to fourteen amino acids [6–9], therefore the AGIA tag (EEAAGIARP), consisting of nine amino acids, can be considered a short peptide tag.

The performance of tag technology strongly depends on the mode of binding between the tag peptide and the antibody. In particular, high specificity and binding affinity are the most important properties for the detection and immobilization of the tag. Anti-AGIA antibody is a rabbit monoclonal antibody with high affinity (K_D_ = 4.90 × 10^−9^ M), which can stably capture an AGIA-tagged target protein (Fig 3B). We demonstrated the strong performance of the AGIA tag system in the detection and immunoprecipitation of AGIA-tagged target proteins (Figs 3 and 4). It is also noteworthy that background levels of the AGIA tag system was extremely low in a variety of cells, including mammalian cultured cells and plant cells (Figs 1B, 3D and 4A).

Current studies on cell biology, which often address complex cellular phenomena, often require the simultaneous detection of multiple target proteins with short tags. Although the FLAG, MYC, HA, and V5 tags are already widely used in cell biology, there is demand for a larger number of powerful tags. As demonstrated in this study, the AGIA tag shows the equivalent performance to, or higher performance than, these conventional tags; therefore, we recommend AGIA tag system as an additional new option.

Cell signaling via posttranslational protein modifications is a key issue in cell biology [34]. Recent proteomics approaches have identified large number of modified cellular proteins with multiple modification sites, especially sites for phosphorylation and ubiquitination [10–13,35,36]. Ser, Thr, and Tyr residues are phosphorylated, and ubiquitination occurs at Lys. If a tag sequence contains these residues, there is a possibility that the fused tag, not the target protein itself, will be modified in the cell. Nevertheless, each of the four widely used tags contains at least one of the four modification residues. Indeed, it was reported that the Tyr residue in the FLAG tag sequence is highly susceptible to sulfation [14]. To avoid the unexpected modification of the target protein, it is preferential to use a fusion tag that does not contain these four amino acids in the sequence. Fortunately, the AGIA tag may provide a solution for this problem because it does not contain these four amino acids.

Purity of a protein sample is essential factor for obtaining accurate biochemical results. Multistep protein purification is widely used to obtain highly purified protein [6,33]. However, a large amount of the starting material is necessary for conventional multistep protocols, which can be a limitation when the target protein is difficult to express. Utilizing a high-performance purification tag is an efficient method for minimizing the purification steps. A purification tag should possess two opposing properties: strong association and rapid dissociation. In general, high-affinity antibodies are not appropriate for protein purification. In fact, the anti-AGIA antibody in the present study showed high affinity against wild-type AGIA sequence and did not release tagged proteins when competed by AGIA peptide treatment (Fig 5C and 5D). However, we found that a single amino acid substitution (E2D) in the AGIA tag facilitated competitive elution of the target protein by adding wild-type peptide. Interestingly, there is not much of a significant difference in kinetic parameters between wild type and AGIA/E2D (Figs 3A and 5E). It can be considered that slightly faster associate constant of wild-type AGIA contributes to inhibit the re-binding of AGIA/E2D tag to anti-AGIA antibody. All three proteins purified by AGIA/E2D in this study were detected as a single band in CBB-stained SDS-PAGE gels (Fig 6A and 6B), and we propose that AGIA/E2D tag is sufficient to purify target proteins using a single step purification protocol under physiological conditions.

Although it is well known that rabbit antibodies have higher affinity than mouse and rat antibodies, development of rabbit monoclonal antibody had been difficult until very recently because of the instability of rabbit hybridomas. Recent technological improvements have allowed production of rabbit mAb [18,19]. Our anti-AGIA antibody (Ra48 mAb) is a rabbit mAb developed using ISAAC technology [19]. In this study, we utilized the advantages of rabbit mAb and developed an AGIA tag system that possesses distinctive features, such as high specificity and low background. To our knowledge, the AGIA tag is the first tag system constructed based on a rabbit mAb and its epitope sequence. Our studies have demonstrated the validity of development additional tag systems based on rabbit mAb.

## Materials and Methods

### General

The following procedures were previously described [37–39]: wheat germ cell-free protein synthesis, split-primer PCR for construction of the DNA templates, parallel syntheses of mRNAs and their translated proteins, protein biotinylation, purification of synthesized proteins, SDS-PAGE, and quantification of proteins using SDS-PAGE and CBB staining by densitometer. All reagents were purchased from Nakarai Tesque (Kyoto, Japan) unless otherwise specified.

### Plasmid Constructions

All primer sequences and plasmids used in this study are listed in S1 Table. pEU-based expression vectors, including pEU-E01-GW, pEU-E01-bls-SrtA-GW, pEU-E01-FLAG-GW, pEU-E01-GW-FLAG, pEU-E01-AGIA-GW, and pEU-E01-GW-AGIA, were used for wheat germ cell-free protein synthesis. pcDNA3.1 and pcDNA3.2/V5-DEST (Thermo Fisher Scientific) are vectors for mammalian cell expression. p35SΩ-GW-NOST was used for protein expression in plant cells [40].

Genes of interest were subcloned using Gateway Technology (Thermo Fisher Scientific). The AGIA tag sequence was inserted at the 5′- or 3′- end of the open reading frame (ORF) by PCR. Deletion and substitution of amino acids in the AGIA sequence were conducted using the PrimeSTAR Mutagenesis Kit (Takara Bio). Human genes (DRD1, MIB2, RelA, MARCH8, OCT4, GATA3 and p53) were obtained from the Mammalian Gene Collection (MGC) full-length cDNA clone set [41]. Mouse Mdm2 was from FANTOM cDNA clones [42]. The cDNA clones of AtGID1A and AtUBQ10 were amplified from RIKEN Arabidopsis full-length cDNA (RAFL) clones [43]. Modification of expression vectors was performed using inverse PCR and In-Fusion cloning kit (Takara Bio).

### Production and Preparation of Anti-AGIA Antibody

cDNAs for the anti-AGIA antibody heavy and light chains [17] were subcloned into the pcDNA3.4 expression vector using PCR and In-Fusion Reaction. Anti-AGIA antibody was expressed using the Expi293F Expression System (Thermo Fisher Scientific) according to the manufacturer’s instruction. The antibody secreted in culture medium was purified by protein G sepharose 4 Fast Flow (GE Healthcare), and then buffer exchange was conducted using a PD-10 column. Purified antibody was frozen and stored at -20°C.

Preparations of antibody conjugated sepharose beads was conducted using NHS-activated Sepharose 4FF (GE Healthcare). Anti-AGIA antibody sepharose was stored at 4°C. HRP-conjugated antibody was prepared using the HRP Conjugation Kit (Abcam). Anti-AGIA antibody-HRP was stored at -30°C.

### Immunoblot Analysis

After separation by SDS-PAGE using e-PAGEL 12.5% or 5–20% (ATTO), proteins were transferred to an Immobilon-P membrane (Millipore) using EzFastBlot (ATTO). After blocking with 5% skim milk solubilized in Tris-buffered saline containing 0.01% Tween20 (TBST) for 1 h, the membrane was treated for 1 h by one of the following primary antibodies at the appropriate concentration: anti-FLAG M2, anti-FLAG-HRP (SIGMA), anti-GST (Molecular Probes), anti-IκBα (Cell Signaling Technology), anti-GFP-HRP (MBL), anti-AGIA, or anti-AGIA-HRP. The membrane was washed 3 times with TBST, and treated with the appropriate secondary antibody diluted 1/10000, including anti-rabbit IgG-HRP and anti-mouse IgG-HRP antibody (GE Healthcare), except when primary antibodies were already conjugated to HRP. After the membrane was washed with TBST three times, the antibody was detected with the Immobilon Western Chemiluminescent HRP Substrate (Millipore) using an ImageQuant LAS 4000 imager (GE Healthcare).

### AlphaScreen

All recombinant proteins were synthesized using a wheat germ cell-free synthesis system. Biotinylation of the biotin ligation site (bls) was conducted enzymatically using BirA biotin ligase [44]. All AlphaScreen reactions were conducted in an Optiplate 384 titer plate (PerkinElmer). AlphaScreen chemiluminescence signal was detected using the EnVision Reader (PerkinElmer).

The analysis of binding between anti-AGIA antibody and the DRD1 fragment was conducted as follows. One μL biotin-SrtA-DRD1 C-terminus fragment and 30 ng anti-AGIA antibody were mixed in 15 μL AlphaScreen buffer containing 100 mM Tris-HCl (pH8.0), 0.01% Tween20, 100 mM NaCl and 1 mg/mL BSA, and incubated at 26°C for 30 min. Subsequently, 10 μL detection mixture containing 0.1 μL streptavidin-conjugated AlphaScreen donor beads and 0.1 μL protein A-conjugated AlphaScreen acceptor beads in AlphaScreen buffer was added to the mixture. After incubation at 26°C for 1 h, AlphaScreen chemiluminescence signal was detected.

The binding analysis between Mdm2 and p53 was performed as follows. One μL biotin-Mdm2 protein was incubated with 1 μL of either FLAG-p53 or AGIA-p53 in 15 μL AlphaScreen buffer at 26°C for 1 h. Then, 10 μL of detection mixture containing detection antibody, anti-FLAG antibody (Sigma, at a final concentration ranging from 8.2 ng/mL to 67.2 μg/mL) or anti-AGIA antibody (from 8.51 ng/mL to 69.8 μg/mL), 0.1 μL streptavidin-conjugated AlphaScreen donor beads, and 0.1 μL protein A-conjugated AlphaScreen acceptor beads was prepared. After 1 h incubation at 26°C, the AlphaScreen chemiluminescence signal was detected.

For DNA-Protein interaction, 1 μL of FLAG- or AGIA tagged GATA3 was mixed with a biotinylated oligonucleotide (10 nM final concentration) in 15 μL AlphaScreen buffer, and incubated at 26°C for 1.5 h. The following oligonucleotides were used: GATA3 consensus DNA, 5′-biotin-CACTTGATAACAGAAAGTGATAACTCT-3′; MT, 5′-biotin-CACTTCTTAACAGAAAGTCTTAACTCT-3′. Subsequently, 10 μL detection mixture, containing either Anti-FLAG M2 antibody (500 ng/mL) or Anti-AGIA antibody (500 ng/mL), 0.1 μL streptavidin-conjugated AlphaScreen donor beads, and 0.1 μL protein A-conjugated AlphaScreen acceptor beads, was added to the reaction and incubated at 26°C for 1 h, and then the AlphaScreen signal was detected. DNA sequence binding to GATA3 was monitored as previously reported [29].

### Mammalian Cell Culture and Protein Expression

HEK293T, HeLa and MCF7 cells were incubated at 37°C under 5% CO_2_ in Dulbecco’s Modified Eagle Medium (DMEM) (Nissui) with 10% fetal bovine serum (Sigma), 2 mM L-glutamine (GIBCO) and antibiotics (100 units/mL penicillin and 100 μg/mL streptomycin) (GIBCO). Huh7 cells were incubated at 37°C under 5% CO_2_ in DMEM-high glucose (Wako) with 10% fetal bovine serum, 1 × MEM NEAA (GIBCO), 1 mM sodium pyruvate and antibiotics (100 units/mL penicillin and 100 μg/mL streptomycin, GIBCO). NIH3T3 cells were incubated at 37°C under 5% CO_2_ in DMEM-high glucose with 10% calf serum (Thermo Fisher Scientific) and antibiotics (100 units/mL penicillin and 100 μg/mL streptomycin). Transfection of pcDNA3.2-DRD1-FLAG into HEK293T cells was conducted using TransIT-LT1 Transfection Reagent (Mirus) according to the manufacturer’s protocol.

### Protein Expressions in Arabidopsis Protoplast

The expression vector containing AGIA-tagged AtGID1A or AtUBQ10 was transfected into Arabidopsis mesophyll protoplasts by polyethylene glycol-mediated DNA transfection as previously described [45]. After overnight incubation, crude extract was obtained by homogenizing protoplasts in protoplast lysis buffer containing 50mM Tris-HCl (pH8.0), 150 mM NaCl, 0.5% sodium deoxycholate, 0.1% SDS, 1.0% Triton X-100, protease inhibitor cocktail (Sigma), and phosphatase inhibitor cocktail (PhosSTOP, Roche).

### Immunostaining

Transfected HEK293T cells were fixed with 4% paraformaldehyde in phosphate-buffered saline (PBS) for 5 min at room temperature, and then permeabilized with 0.5% Triton X-100 in PBS for 5 min. After blocking with 5% calf serum in TBST for 1 h, cells were incubated with anti-AGIA antibody overnight at 4°C. After washing with TBST, cells were incubated with Alexa Flour 488-conjugated anti-rabbit IgG secondary antibody (Molecular Probes) for 1 h at room temperature. Nuclear staining was counterstained with 4,6-diamidino-2-phenylindole (DAPI). After a final wash with TBST, coverslips were mounted with antifade. Images were taken with a confocal laser-scanning microscope (LSM7 Duo, Carl Zeiss).

### Immunoprecipitation

Co-immunoprecipitation analysis of RelA and IκBα was conducted as follows. AGIA-RelA transfected HEK293T cells were lysed with lysis buffer (150 mM NaCl, 25 mM Tris-HCl pH7.5, 1 mM EDTA, 1% Triton X-100) containing proteasome inhibitor and phosphatase inhibitor. Immunoprecipitation of AGIA-tagged RelA was performed by mixing whole-cell lysate with 50 μL anti-AGIA antibody-conjugated sepharose beads by rotation at 4°C overnight. The beads were washed three times with PBS, and immnocomplexes were applied to SDS-PAGE.

AtGID1A-AGIA was expressed in Arabidopsis protoplasts, and the crude protoplast extract was obtained as described above. Immunoprecipitation of AtGID1A-AGIA was performed by the addition of 50 μL anti-AGIA antibody-conjugated sepharose beads into the lysate. The immunoprecipitated protein was visualized by Immunoblotting using anti-AGIA-HRP or anti-AGIA primary antibody and anti-rabbit IgG-HRP secondary antibody.

### Biacore Assay

Biacore experiment was conducted on a Biacore X100 apparatus (GE Healthcare). HBS-EP+ (10 mM Hepes-NaOH (pH 7.4), 150 mM NaCl, 0.05% Tween 20, and 3 mM EDTA) was used as running buffer. The temperature of the flow cells was kept at 25°C.

Kinetics assay was performed by capture method. Protein G was immobilized on a CM5 sensor chip by amine coupling at 6,000 RU to capture antibodies. Analyte, FLAG-GST-AGIA or FLAG-GST-AGIA/E2D protein, was synthesized by cell-free system and purified by glutathione sepharose. Concentration of purified protein was assayed by extinction coefficient method [46] with NanoDrop spectrophotometer (Thermo Fisher Scientific). Extinction coefficient was calculated by using ProParam (http://web.expasy.org/protparam/) [47]. In each cycle, anti-AGIA antibody was captured at 100 RU at 5 μL/min. Then various concentration of analyte was injected at 30 μL/min for 180 sec. After 1,800 sec of dissociation time, the chip surface was regenerated by injecting protein G-regeneration solution containing 10 mM NaOH and 0.5 M NaSCN for 30 sec at 10 μL/min.

Binding and competitive elution of AGIA mutants was using capture method. In each cycle, anti-AGIA antibody was captured on a protein G immobilized sensorchip at 100 RU. Cell-free synthesized FLAG-GST-AGIA protein was diluted 5 times with HBS-EP+ buffer and injected into the flow cells at 10 μL/min for 300 sec. After 1,800 sec of dissociation time, 150 μM AGIA peptide (EKKEEAAGIARPLEK) in Hepes-buffered saline (HBS, 10 mM Hepes-NaOH (pH 7.4), 150 mM NaCl) was injected at 10 μL/min for 300 sec. Finally the sensor chip was regenerated by injecting protein G-regeneration for 30 sec at 10 μL/min.

AGIA/E2D elution condition was examined using capture method. Anti-AGIA antibody was captured on a protein G immobilized sensorchip at 800 RU. Cell-free synthesized FLAG-GST-AGIA/E2D was injected for 60 sec, then each elution solution was injected for 1 min. Finally the chip surface was regenerated by injecting protein G-regeneration solution containing 10 mM NaOH and 0.5M NaSCN for 30 sec at 10 μL/min.

Regeneration test of anti-AGIA antibody was conducted as follows. Anti-AGIA antibody was immobilized on a sensorchip CM5 by amine coupling at 2,000 RU. In each cycle, cell-free synthesized FLAG-GST-AGIA protein was injected for 60 sec at 10 μL/min, then the chip was regenerated by injection 50 mM NaOH for 30 sec.

### Protein Purification

Purification of AGIA-tagged protein by peptide elution was conducted as follows. AGIA/E2D tagged protein was synthesized by wheat germ cell-free synthesis in 6 mL scale, and mixed with 200 μL of anti-AGIA antibody sepharose equilibrated with HBS in advance. The mixture was rotated for 1 h at 4°C. The resin was washed three times with 5 mL HBS, and transferred to spin column (GE Healthcare). The resin was mixed with 200 μL elution buffer containing 150 μM AGIA peptide in HBS, incubated for 1 h at 4μC, and the elution fraction was collected by centrifugation. Elution was repeated three times. Finally, the resin was mixed with 200 μL SDS-PAGE sample buffer and boiled at 99°C for 5 min to analyze protein not eluted by peptide treatment. All fractions were subjected to SDS-PAGE and CBB staining.

TEV-cleavage purification was performed as below. TEV cleavage site was inserted between Venus and AGIA tag by inverse PCR and InFusion cloning kit. Venus-TEV site-AGIA protein was cell-free synthesized at 600 μL scale, and mixed with 20 μL of anti-AGIA antibody sepharose equilibrated by HBS. The mixture was rotated for 1 h, then the resin was collected by centrifugation. After washing with 600 μL of washing buffer containing 10 mM Hepes-NaOH (pH 7.4) and 400 mM NaCl for five times, the resin was mixed with 50 μL of TEV solution containing 1 μL of AcTEV protease (Thermo Fisher Scientific) and 49 μL of HBS. The mixture was rotated for 3 h at 16°C, and then transferred to MicroSpin column (GE Healthcare) and elution fraction was collected. The resin was washed with 50 μL of HBS, and collected as elution fraction 2. Finally, the resin was mixed with 100 μL of SDS-PAGE sample buffer and boiled at 99°C for 10 min. All fractions were subjected to SDS-PAGE and CBB staining.

## Supporting Information

S1 Table List of plasmids and primers. (XLSX)
